# Percutaneous nephrolithotomy: Current concepts

**DOI:** 10.4103/0970-1591.44281

**Published:** 2009

**Authors:** Fabio C. Vicentini, Cristiano Mendes Gomes, Alexandre Danilovic, Elias A. Chedid Neto, Eduardo Mazzucchi, Miguel Srougi

**Affiliations:** Division of Urology, University of Sao Paulo School of Medicine, Sao Paulo, SP, Brazil

**Keywords:** Percutaneous nephrolithotomy, tubeless, kidney stone

## Abstract

Percutaneous nephrolithotomy (PNL) is the procedure of choice for large renal stones. Since its introduction in 1976, many aspects of the operative technique and the endoscopic equipments have had constant evolution, increasing the success rates of the procedure. We performed a literature search using Entrez Pubmed from January 2000 to July 2007 concerning PNL and many aspects related to all steps of the procedure.

We could verify that PNL in supine position has been proved as an acceptable option, but more worldwide experience is necessary. Urologists must be trained to gain their own renal tract access. Minipercutaneous PNL still needs equipments improvements for better results. Tubeless PNL is increasing in popularity and different tract sealants have been studied. Medical prevention is proved to be effective against stone recurrence and should be always used after PNL.

Although the evolution of the technique in the last 20 years, urologists must continue to improve their skills and develop new technologies to offer to the patients more and more a safe and effective option to treat large renal stones.

## INTRODUCTION

Percutaneous nephrolithotomy (PNL) is accepted as the procedure of choice for the treatment of large or complex renal calculi.[1] Since its introduction in 1976,[2] the operative technique and the endoscopic equipments have had constant evolution, increasing the success rates and decreasing complications and morbidity.

Many aspects of the procedure have been debated, while new techniques are constantly introduced. In this review, we discuss important aspects related to the kidney access technique, tract dilation, intraoperative complications, drainage of the collecting system after PNL, treatment of residual stones, and PNL in special cases, such as obese patients, horseshoe kidneys, and stones in caliceal diverticula.

We performed a literature search using Entrez PubMed from January/2000 to July/2007 concerning PNL and aspects related to all steps of the procedure.

## ACCESS TECHNIQUE

### Patient position - Prone vs. supine

Percutaneous nephrolithotomy is usually performed with the patient in prone position through a posterior calyx. This technique is well established, with high rates of success and accepted morbidity.[3] Since 1998 when Valdivia-Uria described the lateral access with the patient in supine position, few groups have used this approach for PNL. The potential advantages of the supine position over the prone position include ease of patient positioning, ability to perform simultaneous PNL and ureteroscopic procedures, better control of the airways, dependent Amplatz sheath drainage facilitating the evacuation of stone fragments.[4] Despite the potential advantages of the supine position, it is not widespread trough the urologic community, perhaps due to the lack of experience and afraid of colonic injuries.

Neto *et al*,[5] have recently demonstrated their experience with 88 PNL in supine position. The procedure was indicated for patients with large or complex staghorn stones located in any part of the kidney, with no exclusion criteria. Ten patients (11.4%) had simultaneous ureteral stones that were treated at the same time without changing patient position. The other patients underwent PNL with a modification to the original position, where the patient is placed supine with the legs extended and the ipsilateral leg crossed over the contralateral leg and using a cushion below the ipsilateral flank to provide a 30° inclination. This position facilitates caliceal puncture, tract dilation, assistant positioning, and decreases potential complications associated with the lithotomy position. The stone clearance rate was 70.5%. No colonic or thoracic injuries occurred, despite of 15 cases accessed through the superior calyx and 4 via supracostal puncture. The blood transfusion rate was 8%.

In another series, Manohar *et al*,[6] describe their experience with PNL in supine position in 62 patients. In this series, the indications were cardiac disease, respiratory problem, obesity (including morbidly obese), and associated upper-ureteral stones. Differently from Neto *et al*, the caliceal access was done under ultrasound guidance instead of fluoroscopy. The rates of blood transfusion, infection, visceral injury, and technical difficulties were 3.2%, 18%, 0%, and 2%, respectively. The clearance stone rate was 95% and the authors commented that in some cases they used flexible nephroscopy to access upper pole stones, which may have contributed for such high stone-free rate. They showed that the supine position can be used even for morbidly obese patients, for whom it may represent an excellent indication due to the difficulties to put these patients in a prone position.

Problems associated to supine PNL are risk of organ injuries, upper pole stone access and necessity of two surgical teams for simultaneous renal and ureteral procedures. Manohar *et al*,[6] use sonography for guide the puncture, so they have a safe window that prevents visceral injuries. Neto *et al*,[5] showed that puncture over the posterior axilar line is safe without the use of sonography, even for supracostal puncture in four cases. Still according this series, in comparison with patients without direct access to upper pole stone no differences were observed in terms of stone clearance rates.

Despite the good results and clear indications for the supine position, there is a lack of randomized studies comparing the supine and prone position. Prone PNL has been performed through the last 25 years, while supine has only about 10 years of experience, so it is expected that urologists prefer for the prone position. Randomized studies with larger series are required to compare both options and define which access technique is more appropriate for each patient.

### Puncture Radiologist vs. urologist

Many urologists do not perform percutaneous access routinely, especially in the United States, where this step of the procedure is frequently performed by the interventional radiologist. Watterson *et al*,[7] retrospectively compared the results of the percutaneous access performed by radiologists and urologists. The primary outcome measures were access-related complications, as bleeding, failure to access, pneumothorax, or other organ injury. The secondary outcome measures were the stone-free rates. Access-related complications and stone-free rates were significantly worse in the radiology access group than in the urology access group. They argue that the use of Seldinger technique combined with antegrade injection of radiopaque contrast to confirm needle placement results in extravasation that ultimately leads to obscuring of the entry calix. Another reason for this worse outcome by radiologists is that they do not have the familiarity with the intricacies of stone removal or placement of multiple tracts for large stone burdens than urologists have.[7]

In a similar study, El-Assmy *et al*,[8] compared the results obtained by radiologists and urologists, and did not find differences in terms of complications and stone-free rates. Both studies indicate that urologists are at least as able as radiologists to safely and effectively obtain percutaneous access for PNL.

To accomplish this, however, it is essential that the urology residency programs are prepared to teach the residents the technique for percutaneous access. It is important to recognize that PNL has a relatively long-learning curve. Two studies evaluated different parameters of the procedure to investigate the learning curve of PNL.[9,10] The main evaluated parameters were operative time, fluoroscopic screening time, and radiation dose. Both studies concluded that the surgical competence in PNL can be reached after 60 procedures while excellence takes around 115 cases. Additionally, they indicated that the most challenging aspect of the surgery is the initial puncture to gain access to the collecting system.[10] The fact that kidney puncture and access is a major step for PNL success highlights the importance of training urologists in this surgical step.

### Puncture techniques

The kidney puncture for gaining access to the collecting system is usually performed under fluoroscopic guidance, after placement of a ureteral catheter through cystoscopy, allowing dilation of the collecting system with saline and contrast media. It requires that the patient is initially placed in a lithotomy position for placement of the ureteral catheter, requiring repositioning to a prone position to perform the puncture and access to the collecting system.

Two different techniques have been suggested. Khan *et al*,[11] reported on the routine use of ureteroscopic-guided percutaneous renal access in 12 patients. The technique consists in gaining access under ureteroscopic vision with the use of the flexible ureteroscope. In this approach, the patient is in the prone position since the beginning of the procedure, precluding positioning changes. They chose the desired calix under ureteroscopic vision, do the pyelography and then perform the puncture to the calix under fluoroscopic view until the tip of the needle is seeing by the ureteroscope, when the guide wire is introduced and the subsequent dilation is performed. Caliceal system access was successfully achieved in all 12 cases, with a stone-free rate of 73%. The authors suggest it may be advantageous for nondilated caliceal systems, complex stone burdens, ectopic or malrotaded kidneys, and for morbidly obese patients, where the tradition puncture is almost always difficulty.

Another study was conducted by Tabibi *et al*,[12] that compared the outcomes of the puncture of the caliceal system with and without retrograde pyelography in 55 patients with opaque renal calculi. For the no retrograde pyelography group, the pyelocalyceal system was approached with the insertion of a small needle toward the opaque stone, without any ureteral catheter insertion. In case of urine aspiration, the contrast media is injected to find out if the direction of the needle in the system is appropriate and dilatation is performed. If the first trial for the system entrance was not successful, the second puncture is performed under the guide of fluoroscopy targeting the stone. Enhancement of the system with intravenous pyelography is used if multiple attempts for the system entrance were not successful. No differences in outcome, infection, operative time, duration of hospital stay, and radiation exposure were observed, indicating that ureteral catheter placement may be precluded. More studies are necessary to establish the role of these two different puncture techniques.

### Miniperc vs. standard PNL

In an effort to reduce morbidity related to PNL in cases with simple or small burden renal calculi and stones located in the lower pole, a PNL performed with smaller caliber instruments was developed and named minipercutaneous nephrolithotomy (miniperc).[13] This procedure is performed through a 13 Fr Amplatz sheath, which might reduce the renal trauma and associated bleeding. It requires the use of a small nephroscope or a rigid or flexible ureteroscope and holmium laser fibers.

Giusti *et al*,[14] retrospectively compared the results of their miniperc series with results from standard PNL and tubeless PNL series (PNL without placement of nephrostomy tube at the end of the procedure) in the treatment of stones <2 cm in diameter. The results were unfavorable to miniperc. The only parameter where miniperc was better was the need for blood transfusions, which was 0% for miniperc, 2.9% for standard PNL and 3.7% for tubeless PNL. In general, miniperc was associated with longer operative time, more pain and hospitalization time than tubeless PNL and a stone-free rate of only 77.5% against 100% and 94% in the tubeless and standard PNL groups, respectively. Monga[15] wrote an editorial comment about this article and affirmed that the major advantage of miniperc is the decrease in blood loss, at the cost of low stone-free rates. He stated that improvements in instrumentation will be needed to increase the success rates of the procedure. Although some groups have described the feasibility and safety of miniperc, no studies have shown a clear benefit of miniperc over standard PNL.[16] Therefore, this is a new technique waiting for new technical developments before it can be offered as a standard technique for the patients.

### Tract dilation - Amplatz vs. Nephromax^®^

The dilation of the tack for creation of the nephrostomy access is a fundamental step of PNL. It is commonly achieved with Amplatz fascial dilators, telescoping metal dilators of Alken or the pneumatic balloon dilators (Nephromax^®^). The balloon dilator seems to be safer, faster, and with reduced X-ray exposition of the patient and of the surgeon.[17] Thus, it is regarded as the gold-standard, but it is associated with a higher cost.

Recently, Al-Kandari *et al*,[18] compared the Amplatz sequential fascial and the balloon dilator in a porcine model. They suggest that the degree of renal trauma induced by each system dilators is comparable, such acutely as chronically, and that the choice of nephrostomy tract dilation method should be determined by urologist personal preference.

Patients with previous open kidney surgeries usually have extensive perirenal fibrosis and in these cases the Alken dilators may be more useful than Amplatz dilator.[19] However, a new balloon dilator, which can achieve 30 atmospheres (X-Force N30^®^), was recently introduced and may prove advantageous in the presence of perirenal fibrosis.[19]

## STONE FRAGMENTATION

### Lithoclast vs. ultrasound vs. Lithoclast ultra

A new combined ultrasound/pneumatic lithotrite device (Lithoclast Ultra^®^; EMS, Nyon, Switzerland) was developed for faster fragmentation of large stones. This new lithotripter is composed of a traditional Lithoclast Master and an ultrasonic device. The 1.0-mm Lithoclast probe is positioned off-center in the hollow 3.3-mm ultrasonic probe and advanced about 1 mm out of the probe, allowing the power of Lithoclast with suction through the ultrasonic probe.[20] The control unit consists of control elements for the Lithoclast and the ultrasound and can be activated separately or in combination. In vitro, it was compared to ultrasound and pneumatic devices by mean time to stone fragmentation and fragment size produced. The combined lithotrite fragmentation time was three times faster than pneumatic and 57% than ultrasound. *In vivo*, it was tested by Hofmann *et al*,[20] in 68 PNL, confirming the feasibility of its use.

Another new intracorporeal lithotrite that was developed is the Cyberwand lithotrite (Cybersonics, Erie, PA, USA).[21] It uses a dual probe design in which two ultrasonic probes are attached to a single-hand piece. The two probes vibrating at different frequencies were constructed to enhance stone comminution. Further fragmentation is thought to be augmented by the ballistic action of the outer probe as it becomes flush with the inner probe from its 1 mm offset position. Cyberwand was compared *in vitro* to the Lithoclast Ultra showing good efficiency to stone penetration and fragmentation. These new devices seem promising for large stones and must have further studies in vivo for define the real role in PNL.

### Evaluation of residual stones

The fate of residual fragments after PNL varies according to their size. It is important to try render the patient stone-free for avoid stone recurrence.

The gold standard method for residual fragments detection is a postoperative noncontrast spiral CT. Another option is a second look procedure, but it is not a desired procedure. Portis *et al*,[22] evaluated the use of high-magnification rotational fluoroscopy combined with aggressive nephroscopy with flexible nephroscope for intraoperative fragment detection during PNL. Their percutaneous nephrolithotomy (PCNL) method involves a team-oriented approach of an urologist, interventional radiologist, and respective support staff, done entirely in an interventional radiology suite. They find that the synergy between the endourologist and uroradiologist allows effective and innovative problem solving in situations requiring improvization. With this technique they successfully detected 100% of fragments >4 mm and 40% of the fragments between 0 and 4 mm. Considering that most of the fragments <4 mm will spontaneously pass, this is a good technique to intraoperatively render the patient stone free. The findings of this study must be compared to the results of the PNL with conventional portable C-arm fluoroscopy in a comparative study to define its real role in treating patients with PNL.

## URINARY TRACT DRAINAGE

### Standard vs. tubeless vs. small bore tubes

After stone fragmentation and extraction, the standard procedure is the placement of a nephrostomy tube. Many groups propose the elimination of the nephrostomy with the advantages of shorter hospitalizations, lack of external drainages tubes, and less postoperative pain. A lack of standardization with regard to kidney drainage and precise indication for the tubeless PNL are an obstacle to widespread utilization of this technique, and its real benefits are not clear.

Tefekli *et al*,[23] prospectively compared the outcomes of standard and tubeless PNL in 38 patients with renal stones. Inclusion criteria were simples, isolated renal pelvis or lower pole caliceal stones, mild or moderate stone burden, and no significant hydronephrosis. They excluded cases that intraoperative complications occurred, operation times exceeding 2 h, cases with more than one puncture, and cases with significant residual stone burden necessitating a second-look PNL. At the end of the procedure, a 14-F nephrostomy tube was placed at least for 48 h in 19 patients, while in the other group only a ureteral catheter was left for 24 h. Comparing the outcomes, the tubeless group was associated with shorter hospital stay (1.6 vs. 2.6 days) and less postoperative analgesia than the nephrostomy group. Mean decrease in serum hemoglobulin level, duration of hematuria, and successful stone clearance was similar in both groups. None of the patients showed evidence of perinephric collection and there was no readmission to the hospital due to postoperative problems. They concluded that tubeless PNL is safe for selected patients with simple renal stones of mild-moderate burden and that mean hospitalization time and analgesia requirement is diminished with this modification.

Shah *et al*,[24] used widespread criteria for indication of tubeless PNL. They excluded only patients that needed more than three percutaneous accesses and presence of significant bleeding that persisted throughout surgery and was not adequately tamponaded by Amplatz sheath. They also left a nephrostomy tube when intraoperatively they verified the presence of a significant residual stone burden necessitating a staged second-look nephroscopy. Otherwise, at the end of the procedure, all patients were left with a double J ureteral catheter. As results, all 45 tubeless PNL in 40 patients were successful, with no significant complications. Two patients required a blood transfusion after surgery. There was no urine leakage or urinoma, or significant chest complication, despite a high index of supracostal punctures. The stone-free status was 87%. They concluded that the tubeless PNL is safe and effective even in patients with solitary kidney, in those with three renal accesses tracts or supracostal accesses, in those with deranged renal values and in those requiring bilateral simultaneous PNL.

Desai *et al*,[25] prospectively compared postoperative outcomes among tubeless, conventional large bore nephrostomy drainage (20 Fr), and small bore nephrostomy drainage (9 Fr) in selected patients following PNL. The tubeless group was associated with the least postoperative pain, urinary leakage, and hospital stay. Small bore nephrostomy drainage was associated with better outcomes than large bore nephrostomy and they concluded that it may be a reasonable option in patients in whom the incidence of stent dysuria is likely to be higher.

In an effort to reduce intraoperative and postoperative bleeding, Jou *et al*,[26] propose eletrocauterization of bleeding points with an electrode probe over the collecting system and access tract. After completion of stone extraction, the irrigation fluid was changed from normal saline to distilled water, and the collecting system and access tract were carefully inspected for bleeding. The bleeding points were usually located just beneath the collecting system and beneath the urothelium and were cauterized with an electrode probe connected to the handpiece of a conventional electric cauterizing device. The purpose of cauterization was to minimize renal hemorrhage, not to stop every bleeding point. They compared two groups, one with 249 patients submitted to eletrocauterization and another with 92 not submitted to that. No statistically significant differences were found in terms of stone burden, operative time, stone-free rate, hospital stay or infection, but the transfusion rate was significantly lower in the eletrocauterization group than in the other group. With this maneuver, 33.7% of the patients submitted to eletrocauterization were rendered free of nephrostomy tubes. They propose that with this technique more patients will be suitable for a tubeless PNL.

Different substances have been used to seal nephrostomy tracts to reduce bleeding and extravasation after tubeless PNL, such as fibrin glue,[27] gelatin matrix[28] and oxidized cellulose (Surgicel^®^),[29] but the real role of these agents in tubeless PNL are still to be defined.

## COMPLICATIONS

Percutaneous nephrolithotomy has acceptably low morbidity. Patient comorbidities and stone burden are the most important predictors for PNL complications.

The AUA Report on the Management of Staghorn Calculi[1] estimates transfusion rates (95% CI) of 18% (14-24%). In a broad group of patients submitted to PNL, transfusion rate is as low as 5.5%[3] Overall significant complications associated with PNL, including acute loss of kidney, colon injury, hydrothorax, perforation, pneumothorax, prolonged leak, sepsis, ureteral stone, and vascular injury, has an estimated rate (95% CI) of 15% (7-27%). Perforation of the collecting system (7.2%) with subsequent urine extravasation caused by a guide wire with straight and hard tip can be avoided by using a J guide wire with a soft and curved tip.[30] Sepsis (0.3-4.7%) is a feared complication.[30] Some investigators suggest administration of oral ciprofloxacin for 1 week before PCNL in patients with stones of ≤20 mm or dilated pelvicalyceal systems to reduce the risk of urosepsis.[31]. Colonic perforation (0.2-0.8%) is rare. Risk factors are left-side procedure, horseshoe kidney, inflated colon, a thin patient, and previous bowel surgery. In case of extraperitoneal perforation, the gastrointestinal tract must keep separated from urinary tract by placing a catheter into the colon and maintaining antibiotics during conservative treatment. On the other hand, an intraperitoneal perforation demands immediate open surgery. Overall risk of pleural injury (0.0-3.1%) is very low, but increases to about 10% if the puncture is above the 12th rib. As such, this puncture should be performed in expiration. PNL mortality risk is up to 0.3%.[30]

The long-term complications reported for PNL include loss of function, perinephric collection, recurrent urinary tract infection, stricturing of the collecting system, urethral stricture, and stone recurrence.

### Hemorrhage

Renal access is the most critical factor for blood loss. Therefore, the urologist must be involved since the first puncture. The ideal access is one that enters a posterior calyx at the fornix, minimizing renal parenchyma traversed, and limiting injury to large vessels. The second most inferior calyx seen on retrograde pyelography is typically posteriorly oriented and is ideal for initial access for most patients.[32] In other words, to avoid extensive angulation and multiple punctures, a flexible nephroscope may be a useful tool.[30]

Conservative measures including adequate hydration, prevention of hypothermia, clamping the nephrostomy tube, diuretics, and hemostatic drugs are adequate to treat mild bleeding. Moderate hemorrhage demands blood transfusion in addition to conservative measures. If hemodynamic instability is noted, superselective renal angiography to identify the site and type of vascular injury leading to severe bleeding is the treatment of choice. Significant risk factors for severe bleeding are upper caliceal puncture, solitary kidney, staghorn stone, multiple punctures, and inexperienced surgeon.[3] Superselective renal angiography is required in 1% of procedures. Pseudoaneurysm, arteriovenous fistula, and arterial laceration are the most common lesion revealed by renal angiography. Severe bleeding can be controlled in up to 92% of patients with superselective embolization.[3] In the event of embolization fail, the patient must be submitted to urgent open exploration with a high probability of nephrectomy. Sometimes, bleeding occurs after discharge home. Medium time for the onset of postoperative bleeding due to pseudoaneurysm or arteriovenous fistulas occurs at 8 days, but patients can present months after PNL. These patients should be managed with embolization.[32]

## SPECIAL CASES

### Diverticular calculi

Caliceal diverticula are congenital, nonsecretory, urothelium-lined cavities within the renal parenchyma that communicate with the caliceal fornix through a diverticular neck. Management of diverticular calculi involves stone removal and treatment of the diverticular cavity. This can be achieved with PNL and diverticulum fulguration with 85.7% of stone-free rate and 87.5% of diverticulum obliterated.[33] Fulguration of the diverticular lining is performed with a 24-Fr resectoscope with a rollerball electrode. Diverticulum treatment can also be achieved by creating a large communication to the collecting system to promote drainage and prevent urinary stasis. Neoinfundibulotomy require the placement of a nephrostomy tube for a prolonged period. This technique has a stone-free rate of 80-93% and 63-76% of diverticulum obliterated.[34,35]

### Elderly patient

There is no substantial data on this matter. At this point, we do not have data that assume a higher risk of PNL in subjects over the age of 70 years. On the contrary, it seems to be a safe procedure and may achieve a stone-free rate up to 70.8% for all types of stones combined in a retrospective study.[36] Diabetes was the only independent risk factor associated with increased overall operative risk.[36] In this study, postoperative fever was the most common complication (19.6%) followed by intraoperative bleeding (7.1%) with a transfusion rate of 7.7%. They propose a rigorous preoperative medical evaluation and control of the comorbidities for reduce the operative risk.

### Morbidly obese

Several authors advocate that morbidly obese patients have a similar stone-free rate and complication rate compared to no obese patients based on retrospective studies.[37,38] Supine PNL approach should be considered when simultaneous management of renal and ureteral stones is an issue.[6]

### Prevention of recurrence of nephrolithiasis

Specific dietary measures with repeated dietary counseling and metabolic monitoring are more effective in reducing stone recurrence rates than non-specific measures and limited follow-up (7% vs. 23%).[39]

Metabolic evaluation and aggressive medical management can control stone formation in patients with or without residual stone fragments after PNL. Medical management should be instituted in patients following PNL without regard to stone-free status.[40] Kang *et al*, studied 70 patients placed into four groups following PNL, that is stone-free or residual fragments, who underwent or did not undergo medical therapy. Selective medical therapy significantly decreased stone formation rates in the stone-free (0.67 stones per patient per year vs. 0.02) and residual fragment groups (0.67 stones per patient per year vs. 0.02; *P* < 0.0001), and remission was observed in a higher proportion of patients in the medically treated stone-free and residual fragment groups (87% and 77%) when compared to the same groups without medical therapy (29% and 21%; *P* < 0.0001). They concluded that even patients with residual stones clear benefits of selective medical therapy after PNL.

## CONCLUSIONS

Percutaneous nephrolithotomy technique is in constant evolution. Supine position has been proved as an acceptable option. Urologists must be trained to gain their own renal tract access. The miniperc PNL still needs equipments improvements for better results. Tubeless PNL is increasing in popularity and different tract sealants have been studied. Medical prevention is proved to be effective against stone recurrence and should be always used after PNL. Although the evolution of the technique in the last 20 years, urologists must continue to improve their skills and develop new technologies to offer to the patients more and more a safe and effective option to treat large renal stones.
